# Moral Disengagement in Adolescent Offenders: Its Relationship with Antisocial Behavior and Its Presence in Offenders of the Law and School Norms

**DOI:** 10.3390/children11010070

**Published:** 2024-01-08

**Authors:** Daniela Agudelo Rico, Carolina Panesso Giraldo, Joan Sebastian Arbeláez Caro, Germán Cabrera Gutiérrez, Valeria Isaac, María Josefina Escobar, Eduar Herrera

**Affiliations:** 1 Universidad Icesi, Departamento de Estudios Psicológicos, Cali 760031, Colombia; 2 Center for Social and Cognitive Neuroscience, School of Psychology, Universidad Adolfo Ibáñez, Santiago 2580335, Chile; carolina.panesso@edu.uai.cl (C.P.G.); misaac@alumnos.uai.cl (V.I.); 3 Facultad de Psicología, Universidad de San Buenaventura Medellín, Medellín 050035, Colombia; 4 Facultad de Ciencias Humanas y de la Educación, Corporación Universitaria Empresarial Alexander Von Humboldt, Armenia 63001, Colombia; gcabrera89@cue.edu.co

**Keywords:** antisocial behavior, moral disengagement, adolescent school norm offenders, adolescent legal offenders, adolescent lawbreakers, aggression

## Abstract

This study focuses on understanding the relationship between moral disengagement mechanisms in adolescents who engage in law-breaking activities and those who violate school norms. To do so, we administered the Mechanisms of Moral Disengagement Scale (MMDS), which evaluates moral justification, euphemistic labeling, advantageous comparison, deflection of responsibility, diffusion of responsibility, distortion of consequences, dehumanization, and attribution of blame, to 366 adolescents (60.1% males (*n* = 220) and 39.9% females (*n* = 146)). Our results confirmed the hypothesis that law-breaking adolescents presented a higher degree of moral disengagement than those adolescents who violate school norms. Additionally, we found that adolescents who violated school norms displayed significantly higher levels of dehumanization than the controls, and law-breaking adolescents obtained the highest score in this domain. Our findings allow us to suggest that the presence of the dehumanization mechanism in adolescents who violate school norms could be used as an early indicator of the emergence of antisocial behaviors, since this was the only component of moral disengagement that significantly differentiated this group from the controls in the study.

## 1. Introduction

Each community and society generate their own conditions to establish norms with the objective of maintaining harmony and social order. In turn, subjects learn and adopt moral principles that act as regulators of behavior [1] for the common wellbeing. Special attention is paid to those individuals who challenge the established social order by means of behaviors that transgress the norms, and in this way, the understanding of how and why such transgression is reached, and how this affects the social balance, has been an object of interest in the scientific community.

Behavioral problems encompass a series of actions that may transgress and oppose socially accepted norms and values to more severe actions such as sexual offenses, robbery, and assault [1,2,3,4,5,6,7]. Nonetheless, behavioral problems that are associated with difficulties in conforming to social norms and values should be distinguished from antisocial disorder, which refers to a clinical diagnosis with a defined set of symptoms and is not a target population in this study [8].

In recent decades, the moral disengagement theory introduced by Bandura [9] has been widely used as a theoretical model to explain antisocial behavior. Different studies have evidenced a relation between the use of moral disengagement and norm-transgressive behaviors in adolescents [10,11,12,13,14,15,16,17,18,19], where moral disengagement could serve as an important predictor of antisocial behavior in youth [8,20,21].

Moral disengagement describes the process through which people justify or minimize the severity of their behavior, despite it being morally questionable, harmful, and damaging to others. This dynamic enables the subject to act without experiencing remorse, discomfort, or guilt for his or her actions. Subjects develop their own interpretations of the performed behavior, distorting it into something that is acceptable and devoid of its original negative qualities [1]. Moral disengagement is supported by several mechanisms that allow the person to emotionally and cognitively disengage from the consequences of his or her behavior [9]. These mechanisms are classified into four key aspects: (1) disengagement from the harmful behavior and denying participation in the act, which includes mechanisms of moral justification, advantageous comparison, and euphemistic language; (2) the reduction in the degree of responsibility as the perpetrator of the harm, which includes the mechanisms of displacement and diffusion of responsibility; (3) the misrepresentation of the repercussions and effects of violent or immoral actions, where the mechanism of distortion of the consequences is located; (4) and finally, the blaming and objectification of the victim, where one makes use of the mechanisms of attribution of blame and dehumanization [1,9,22,23]. In this sense, moral disengagement allows adolescents to not feel guilty for their own actions [8,24], justify antisocial behaviors, stop punishing themselves [25], preserve their positive self-image, maintain their social position, and avoid the stigma that society associates with legal offenses [26]. 

In recent years, moral disengagement in the adolescent population has been studied, especially in terms of delinquent behavior [16,19,27,28] the violence of armed groups outside the law [13,25,29,30,31], bullying [32,33,34], cyberbullying [35,36,37,38,39], video games [40,41,42,43], moral disengagement and its relation to empathy [44,45], psychopathy [18,46], moral disengagement and civic norms [47], and sex offenders [48,49,50], among others, evidencing through varied research the importance of understanding moral disengagement to explain the social behaviors that are involved in moral decision making in different contexts [31].

Regarding adolescent offenders, Gomez and Durán [13] sought to examine how moral disengagement influences behavior and the justification of actions in victims of armed conflict, delinquents, and students. It was found that adolescent legal offenders presented the highest scores in the different sociocognitive mechanisms of moral disengagement compared with the other groups of adolescents. Likewise, Canchila et al. [51] investigated the use of moral disengagement mechanisms in adolescents and found that in early adolescence, the most commonly used mechanisms are responsibility transfer, diffusion of responsibility, distortion of consequences, and victim blaming.

D’Urso et al. [49] analyzed the mechanisms of moral disengagement and the relationship with the use of psychoactive drugs during adolescence in 49 incarcerated offenders in Italy, finding that young people with a frequent use of these drugs reported higher levels of general moral disengagement, dehumanization of victims, and advantageous comparison compared to adult offenders. Brugués and Caparrós [52] examined moral disengagement mechanisms in relation to antisocial behaviors and recidivism. The results indicated that recidivists scored higher on all moral disengagement mechanisms. In particular, advantageous comparison and dehumanization were the moral disengagement mechanisms that were most strongly associated with this phenomenon. The study by Gomez et al. [45] investigated the relationship between callous–unemotional traits, moral disengagement mechanisms, and empathy capacity in adolescent offenders. A significant connection was found between the lack of empathy in juvenile delinquents and their tendency to use moral disengagement mechanisms. It was also found that as the level of empathy of offenders increased, their tendency to use these moral disengagement mechanisms decreased. Barrett [53] and Walters [28] found that adolescents who presented serious behavioral difficulties, as well as those who participated in delinquent group activities, showed a higher probability of persisting in antisocial behaviors. Concha-Salgado [54] adapted and validated Bandura’s Moral Disengagement Scale in Chilean adolescents to evaluate its relationship with aggressive and transgressive behaviors. Their findings showed a connection between moral disengagement and adolescents’ participation in delinquent and violent behaviors. In addition, it was observed that certain sociodemographic factors, such as age, sex, and socioeconomic status (SES), may influence the relationship between moral disengagement and transgressive behaviors, suggesting that these individual characteristics could have a significant impact on how the association between moral disengagement and delinquent or violent behavior manifests itself in the studied population. In addition, moral disengagement was found to be a self-regulatory cognitive process that adolescents use when confronted with transgressive and aggressive behaviors. The scale was successfully adapted, and moral disengagement was found to be a significant predictor of transgressive behaviors in Chilean adolescents. Finally, Cabrera et al. [8] analyzed whether moral disengagement can be a predictor of antisocial behavior in adolescents that are in conflict with the law. The results showed that moral disengagement is a significant factor in antisocial behavior in these adolescents. In addition, a relationship was found between moral disengagement and the age of onset of delinquent behavior, time of internment, and educational level.

The literature highlights the importance of research focusing on adolescent antisocial behavior due to the significant biological and psychological changes that occur during this stage, making this a critical and highly susceptible period for the manifestation of these behaviors [55,56,57,58]. Changes in neural circuits that are present in brain areas which are responsible for reward processing and motivation during adolescence have been related to behavioral changes observed in this developmental period [59,60] These changes are characterized by a decrease in the risk perception and assessment of the consequences of actions, which could be linked to a greater inclination toward the pursuit of immediate gratification [57,61], which in turn may contribute to a decreased capacity for emotional self-regulation [62,63]. In addition, young people may be more susceptible to negative environmental influences [64] and, at times, defy social and moral norms, generating the manifestation of transgressive behaviors [32,45,65,66]. A lack of emotional self-regulation may contribute to the persistence of antisocial behaviors by influencing the development and maintenance of moral disengagement, which plays an important role in the adoption and justification of such behaviors [54,67,68,69]. Given that criminal trajectories usually begin during adolescence [70], the persistence of antisocial behaviors in young people represents a growing threat to society. According to available figures, in Colombia, there are a significant number of young people who are part of the criminal liability system who are involved in criminal activities, reaching 8400 in 2022. Ninety percent of these youths are male, and they mostly come from vulnerable backgrounds, as reported by the Colombian Institute of Family Welfare. In addition, about 38.7% have problems related to drug use, and 9% have mental health disorders, according to the Ministry of Health.

Some authors have pointed out the need to study moral disengagement in different contexts and specific situations. It is important to note that in general terms, most studies have evaluated the relationship between moral disengagement and antisocial behavior in adolescents at a general level, but there are no scientific sources showing the influence of moral disengagement mechanisms and their link with adolescent lawbreakers and school norm violators. Therefore, the present study aims to address this problem, contributing to the current understanding of the topic of interest and contributing new perspectives to the existing knowledge base.

To achieve this, we sought to determine whether the mechanisms of moral disengagement were more present in adolescents linked to the criminal liability system in Colombia and in offenders of school norms compared to a control group. These findings could provide evidence of the early indicators of the emergence of antisocial behaviors and, consequently, could lead to the creation of more effective intervention programs to prevent future delinquent behaviors.

Based on previous research and existing theories such as moral disengagement and antisocial behavior, we hypothesize in this study that adolescents in the criminal liability system would have or present higher scores on disengagement mechanisms, and that these in turn would be lower than adolescents that are referred for school counseling and controls (the latter having lower scores). Data were collected by administering the Moral Disengagement Mechanisms Scale (MMDS) and subsequently analyzed using a multinomial logistic regression approach.

## 2. Methods

### 2.1. Design

This study is quantitative with a nonexperimental design of cross-sectional temporality. Its scope is correlational, seeking to analyze the association between the different variables in this study, which are the mechanisms of moral disengagement and antisocial behavior in adolescents. Given the nature of the present study, considering a medium effect size (d = 0.5), a statistical power of 80%, alpha = 0.05, and one-tailed hypothesis, a minimum of 100 cases per group is required, and sample size calculation was conducted using GPower.

### 2.2. Participants

The study included a total sample of 366 adolescents, of whom 60.1% were male (*n* = 220) and 39.9% female (*n* = 146), between the ages of 14 and 17 with an average age of 15 years (SD = 1.02) and a low SES level (M = 2.19; SD = 1.03).

Participants in this study were divided into three groups: the first group was selected from the Department of Quindío and included 122 adolescent legal offenders, sanctioned by the competent authority (Judge) or referred by family ombudsmen of the Criminal Liability System for Adolescents (CRSA) in the CIFW with processes of restoration of rights (males *n* = 110; females *n* = 12; S.D. = 0.88). The second and third groups of adolescents were recruited from educational institutions of the city. The second group, the school norm violators (SNVs), included 122 adolescent students known to commit disciplinary and/or coexistence transgressions of school norms (males *n* = 62; females *n* = 60; S.D. = 1.00). Lastly, the control group consisted of 122 adolescent students known to respect school norms (males *n* = 48; females *n* = 74; S.D. = 0.65). The sample was selected through nonprobabilistic purposive sampling, where sociodemographic variables such as sex and SES were considered. The size sample was according to the number of adolescents found in the criminal liability system at the time of assessment, and we intentionally assessed SES level, as we wanted to equate the socioeconomic context of youth involved in the criminal justice system with youth from schools where participants were recruited for the study.

To participate in this study, adolescents were required to show a signed consent from their parent/guardian. Adolescents who were selected for the study did not present any intellectual disability or any other condition that might affect their ability to complete the tests, including being under the influence of psychoactive substances or medications that would affect their ability to respond. In addition, participants with additional conditions such as mental retardation, autism spectrum disorder, and other disabilities that might limit participation in the research were excluded. It should be noted that these conditions were not directly diagnosed by us, but we investigated whether participants had received such a diagnosis through information provided by their parents.

The intention to select three samples of adolescents responds to the need to isolate the variable of: “antisocial behavior” and “criminal behavior”, since the adolescents associated with the SRPA have committed an offense according to the Colombian Juvenile Code and their behavior was considered “criminal”, but the adolescents who violated school rules have not committed an offense per se, but rather “antisocial” behavior, while the control group has not committed either of these two behaviors.

### 2.3. Ethical Considerations

The present study was approved by the Bioethics Committee of the University of San Buenaventura, Medellín, on 24 August 2016. Participants and their families were informed about on secrecy and handling of information, right to nonparticipation, right to return of information, and accompaniment by the research team. The risk of the research was reported as a minimum considering article 11 of resolution 008430 of the Colombian Ministry of Health.

### 2.4. Instruments

**The Moral Disengagement Mechanisms Scale (MMDS)** developed by Bandura [1] is a Likert-type scale that aims to study the level of moral disengagement in a weighted score and according to the eight mechanisms proposed byBandura [9]. The items point to the willingness of participants to resort to moral justification, euphemistic labeling, advantageous comparison, displacement and diffusion of responsibility, distortion of consequences, dehumanization, and attribution of blame for different forms of transgressive behavior [1]. The scale for measuring the mechanisms of moral disengagement contains a total of 32 items with five response possibilities, “not at all agree”, “slightly agree”, “quite agree”, “strongly agree”, and “absolutely agree”, with a high reliability index of α: 0.93 [71], α: 0.76 [72]. The Spanish version of Rubio-Garay et al. [23] was used, which has an overall α = 0.87 and maintains the original factors considered by Bandura [1]. This version has been used in different studies in Colombian adolescent populations [13,25,30,31]. In the present study, the scale expressed an α: 0.92.

**The characterization form**, a questionnaire designed specifically for this study, collects sociodemographic and socioeconomic information about the juvenile, as well as relevant details about the offense committed by the juvenile. This characterization form is not a previously standardized instrument but was created specifically for this research. It covers key aspects such as the adolescent’s gender, type of crime, educational level, socioeconomic level, family typology (traditional, single-parent, or extended), and the adolescent’s admission to and involvement in the SRPA.

### 2.5. Procedure

After obtaining the approval of the corresponding ethical bodies, the families and adolescents were provided with an explanation of the ethical parameters and implications of the study. Parents or legal guardians were asked to sign the informed consent form, and the adolescents were asked to give their assent. Once consent was obtained, adolescents were instructed to read each question carefully and respond based on their experience. To avoid any possible bias, the order of the instruments was randomized among participants. The application of the questionnaires was carried out in a single 40 min session in the classrooms of the corresponding institutions. Confidentiality and anonymity of the information provided were assured. Finally, the results of the evaluation were digitized and coded in an Excel data matrix for subsequent analysis.

### 2.6. Analysis Plan

Descriptive analyses were carried out to obtain averages and standard deviation for each variable, followed by a correlation matrix of the study variables. Finally, a multinomial logistic regression model was applied, as well as a one-factor ANOVA model with comparisons between groups. All these analyses were carried out in the statistical program Rstudio, V.4.1.1.

## 3. Results

The findings of this study are structured into two main sections. The first section presents the descriptive and correlational results of the study variables. The final section presents the results that were obtained by multinomial logistic regression analysis. The descriptive statistics, where the mean values obtained by the study participants in each of the variables can be observed, are shown in Table 1, Table 2 and Table 3. The bivariate correlations are shown in Table 4 and the multinomial logistic regression in Table 5.

Regarding the type of crime, a total of 136 participants (37.2%) were linked to the SRPA, of which 95 were linked only once (26%), 18 were linked twice (4.9%), 4 were linked three times (1.1%), and 4 participants were linked more than four times (1.1%).

The bivariate correlations showed moderate and positive correlations between the scales that made up the variable Moral Disengagement Mechanisms Scale (MMDS). In relation to the participant’s age, a small and significant correlation was observed with the average of the Moral Disengagement Scale (r = 0.17, *p* < 0.001). Finally, the only variables that had a small and significant correlation with the variable stratum were the subscales of attribution of blame (r = −0.15, *p* < 0.01), dehumanization (r = −0.10, *p* < 0.05), and age (r = −0.23, *p* < 0.01).

A multinomial logistic regression was performed to predict membership of the three groups: the penal system group (CRSA), the group of school norm violators (SNVs), and a control group. The predictors included in the model were sex, age, SES, and sum (of the general group).

Compared to the control group, sex showed an odds ratio of 1.84 (95% CI: 0.98–3.46, *p* = 0.05) for the SNVs, and a significantly higher odds ratio of 13.1 (95% CI: 5.48–31.6, *p* < 0.001) for the CRSA. Age also showed a significant relationship with both groups, with an odds ratio of 2.43 (95% CI: 1.69–3.51, *p* < 0.001) for the SNV group and 4.78 (95% CI: 3.08–7.41, *p* < 0.001) for the CRSA group. SES showed a negative and significant association in both cases, with an odds ratio of 0.43 (95% CI: 0.32–0.59, *p* < 0.001) for the SNV group and 0.30 (95% CI: 0.20–0.46, *p* < 0.001) for the CRSA group.

The variable MDDS Scale, which is the average of the entire scale measuring moral disengagement mechanisms, on the other hand, did not show a significant association with the SNV group (odds ratio = 1.015, 95% CI: 0.991–1.03868, *p* = 0.223); however, it did show a positive and significant relationship with the CRSA group (odds ratio = 1.038, 95% CI: 1.012–1.06469, *p* = 0.005).

In summary, differences in sex, age, and SES were significant predictors for membership in the SNV and CRSA groups compared to the control group, while the MDDS scale variable, which is an average measure of moral disengagement mechanisms, only showed a significant relationship with the CRSA.

Finally, we ran a one-factor ANOVA model; considering that the variables did not have a normal distribution, we used the ANOVA (Kruskal–Wallis) (Table 6) and, subsequently, the Dwass–Steel–Critchlow–Fligner two-by-two comparisons (Table 7), finding that the scales, where there were statistically significant differences between the group belonging to the CRSA and the control group, were as follows: total scale (W = 6.79; *p* = <0.001), moral justification (W = 7.08; *p* = <0.001), advantageous comparison (W = 6.83; *p* = <0.001), displacement of responsibility (W = 4.83; *p* = <0.01), distortion of consequences (W = 3.49; *p* = <0.05), attribution of blame (W = 6.74; *p* = <0.05), and dehumanization (W = 5.50; *p* = <0.001). Between the SNV and the CRSA groups, the scales, where there were differences, were as follows: total scale (W = 4.27; *p* = <0.01), moral justification (W = 4.91; *p* = <0.01), advantageous comparison (W = 5.52; *p* = <0.001), attribution of blame (W = 4.30; *p* = <0.01), and dehumanization (W = 3.66; *p* = <0.05).

## 4. Discussion

The purpose of this study was to determine the degree of relationship between moral disengagement mechanisms in adolescent legal offenders (CRSA) and school norm offenders (SNVs). It was hypothesized that adolescents involved in the criminal liability system would score higher on moral disengagement mechanisms compared to adolescent school norm offenders and the controls. The latter were expected to have lower scores on these mechanisms.

Consistent with our hypothesis, the results of the present study indicated that there are indeed significant differences in the various evaluated domains. In general, the results show that adolescent offenders score significantly higher on the Moral Disengagement Scale than adolescent offenders of the school norms and the control group, with adolescent legal offenders presenting greater moral disengagement. This finding is consistent with the current research [13,25,29,31,32,48,49,50,52,73], which points out that adolescents who have committed antisocial behaviors scored higher in all the moral disengagement mechanisms of those assessed.

On the other hand, it was observed that the CRSA group exhibited higher levels of dehumanization compared to the SNV and control groups. However, it should be noted that the SNV group also showed higher levels of dehumanization compared to the control group. Dehumanization causes people to objectify their victims, denying their human attributes to justify harmful or immoral actions against them [9]. 

In agreement with previous studies [13], we found a significant difference in the dehumanization mechanism between adolescents that were disengaged from armed groups and lawbreakers, with the latter presenting higher averages. In this sense, the fact that the dehumanization mechanism was the only component differentiating the moral disengagement between these two groups means that it could be used to predict the emergence of antisocial behaviors in adolescents deviating from the school norms. According to [74], school violence is associated with dehumanization, and individuals who present higher levels of dehumanization are more likely to be aggressors.

Finally, continuing with the analysis of the most important results, differentiating elements were found in the study, mainly in sex and the SES level variable. Sex was a differentiating factor [8,13,16,34,45,73,75,76]. As indicated by the present research, significant differences were found in moral disengagement according to sex, mainly because it is men who have a greater proclivity to present antisocial and criminal behaviors. According to data from the CIFW (2018), in Colombia, men are the ones who present a higher prevalence of delinquent behaviors and sanctions. This is related to studies that have shown that men have a greater tendency to use moral disengagement mechanisms compared to women, which has been associated with a higher prevalence of disruptive behavior problems [75]. 

In this study, significant variations in moral disengagement were also identified in relation to SES. These differences suggest that SES could be a predictive and causal factor in the prevalence of moral disengagement and, therefore, in norm-breaking behaviors in adolescents [16]. Previous evidence has shown that SES could be a risk factor for adolescent behaviors, which translates into a higher probability of aggressive behaviors, drug use, and participation in criminal activities, which could lead to a higher probability of involvement in antisocial behaviors [27,74,77,78]. According to [78], delinquency is a complex phenomenon that originates in a process of social maladjustment involving multiple factors. In this process, various elements intervene and interact with each other, generating a multidimensional dynamic of the subjects. In this sense, exposure to certain contexts could trigger moral disengagement and, therefore, become a risk factor for antisocial behavior.

There are several limitations that should be considered when interpreting the findings of this study. The results obtained in this research are based on self-reported measures, which could generate biases and errors in the interpretation of the results [13]. Participants’ responses may vary according to their understanding or interpretation of the questions, and the different ways in which people fill out the rating scales may influence the scores obtained. Therefore, differences in scores among participants may not reflect only what the questionnaire was designed to measure. In addition, the use of self-reported measures may be affected by the social desirability of the participants, which may influence the reliability of the scores [54]. Similarly, it should be noted that the cross-sectional design used in this study does not allow us to establish causal relationships between the analyzed variables. In order to be able to make causal inferences, it would be necessary to have longitudinal designs that allow us to follow the participants over time. In addition, it is important to keep in mind that the use of a cross-sectional design limits our ability to infer causal relationships and to assess whether the associations between moral disengagement mechanisms and violent antisocial behavior are stable over time. Furthermore, we cannot determine whether one variable longitudinally influences the others in the model [34,54]. It is important to mention as a limitation of this study the use of only one instrument to observe the variable of interest (moral disengagement). Future studies should consider other instruments to evaluate the relationship between variables (such as emotional disturbances or psychiatric disorders) or to predict which other factors might be interacting with moral disengagement in adolescents.

In this study, we analyzed and compared the main findings of various research studies that focused on the phenomenon of delinquency, which have been discussed in order to generate a constructive dialogue and raise new questions and challenges. These results allow us to identify common patterns in delinquent behavior in the context studied and contribute to a better understanding of the dynamics underlying antisocial behavior. In most of the studies conducted in this field of research, the relationship between adolescent offenders and moral disengagement mechanisms has been limited to examining criminal behavior indiscriminately [18], where emphasis has been placed only on adolescents that are in conflict with the law [8], as well as victims of an armed conflict, delinquents, and the student population in general [13,25,30,31,32]. 

Until now, no research involving adolescents who have transgressed school norms had been carried out with these population groups. The novelty presented in this study is that the relationship analyzed has not been previously addressed specifically among these three different groups of adolescents, which makes it possible to identify the personality traits and cognitive mechanisms that are associated with delinquent behavior in these adolescents. By analyzing these associations, we can obtain valuable information about risk factors that could predict future delinquency among young school norm offenders. Likewise, the results obtained in this study are important not only for their theoretical value in deepening our understanding of the moral behavior of adolescents but also for their social relevance in the development of programs based on an interdisciplinary approach to juvenile delinquency, as well as in educational intervention. Schools constitute a key space to promote prosocial behaviors and reduce interpersonal violence, which is often frequent during adolescence [79]. 

Although there is research that supports the results of this study, it is important to highlight that there are few studies in Latin America and particularly in Colombia that focus on exploring the relationship between moral disengagement and disruptive behaviors in adolescents in a situation of psychosocial vulnerability in the classroom [13,32]. It is widely recognized that bullying in schools is a significant problem in the country; Colombia is in second place among the ten Latin American countries with the highest cases of bullying and ranks tenth in bullying cases worldwide [80]; one in five students (32%) report having been victims of bullying, with a total of 8981 serious cases of bullying occurring between 2020 and 2021 [81]. Therefore, in the educational setting, it is pertinent to incorporate the Moral Disengagement Scale in comprehensive prevention programs that simultaneously address various risk factors for violent behavior in the school environment.

It would be interesting for future research to focus on comparatively analyzing not only the mechanisms that are associated with moral disengagement but also the emotional aspects that are present in these three population groups.

We also recommend a more detailed analysis of other variables such as sociodemographic and psychosocial variables and consumption of psychoactive substances in adolescents with diverse characteristics in order to obtain a more complete understanding of the factors that influence their behavior and to evaluate the influence of other contexts; in this sense, what other individual circumstances could influence the degree of moral disengagement observed in these adolescents? How could the presence of these mechanisms of moral disengagement vary in these young people from different cultures or socioeconomic levels? Likewise, as mentioned by Gómez and Durán [13], the findings of this type of study suggest the need for further research to better understand the factors that influence these differences and thus obtain more solid evidence on the subject. Finally, in future research, it would be advisable to obtain information from external sources, among families, educators, and peers, to complement the data obtained through self-reports and thus have a more complete and accurate view of the studied phenomenon. This would make it possible to contrast the information provided by the participants with that of people who are close to them and obtain a more objective evaluation of the studied behaviors and traits.

## Figures and Tables

**Table 1:** Descriptive statistics.

Sociodemographic Data	Total Group		CRSA Group		SNV Group		Control Group	
	*n* = 366		*n* = 122		*n* = 122		*n* = 122	
	M	SD	M	SD	M	SD	M	SD
Age	15.56	1.02	16.24	0.88	15.56	1	14.89	0.65
SES Level	2.19	1.03	1.79	0.83	2	1.04	2.89	0.93
Educational Level	8	1.50	7	1.94	9	0.79	9	0.49
MMDS Scale	56.0	17.2	63.6	23.3	53.8	11.2	50.6	11.2
Moral Justification	7.62	3.01	8.97	3.72	7.29	2.48	6.61	2.10
Euphemistic Language	7.07	2.57	7.67	3.39	6.76	1.97	6.79	2.01
Advantageous Comparison	5.68	2.56	6.84	3.49	5.19	1.62	5.01	1.69
Displacement of Responsibility	7.53	3.15	8.47	3.84	7.32	2.51	6.80	2.72
Diffusion of Responsibility	7.99	3.32	8.52	4.19	7.97	2.66	7.47	2.84
Distortion of Consequences	7.57	2.95	8.31	3.61	7.35	2.48	7.06	2.47
Attribution of Blame	6.54	2.69	7.81	3.57	6.18	1.86	5.62	1.74
Dehumanization	5.97	2.72	6.97	3.53	5.74	2.13	5.20	1.92

M: Mean; SD: Standard deviation.

**Table 2:** Descriptive statistics of participants’ sex.

Sociodemographic Data	Total Group		CRSA Group		SNV Group		Control Grupo	
	*n* = 366		*n* = 122		*n* = 122		*n* = 122	
	N	%	N	%	N	%	N	%
Male	220	60.1	110	90.2	62	50.8	48	39.3

CRSA group: Adolescents belonging to the criminal liability system; SNV group: Adolescent offenders of school norms.

**Table 3:** Type of crime committed by the CRSA group.

Crime	N	%
Homicide	7	5.74
Theft	30	24.59
Trafficking in narcotics	29	23.77
Conspiracy to commit a crime	5	4.10
Sexual abuse	4	3.28
Violence against public officials	2	1.64
Personal injury	5	4.10
Extortion	3	2.46
Kidnapping	0	0.00
Damage to the property of others	2	1.64
Family violence	17	13.93
Attempted homicide	14	11.48
Weapons possession	4	3.28

**Table 4:** Correlation matrix.

	1	2	3	4	5	6	7	8	9	10
1. MMDS Scale	-									
2. Moral Justification	0.75 ***	-								
3. Euphemistic Language	0.68 ***	0.53 ***	-							
4. Advantageous Comparison	0.68 ***	0.45 ***	0.44 ***	-						
5. Displacement Responsibility	0.72 ***	0.39 ***	0.44 ***	0.51 ***	-					
6. Diffusion of Responsibility	0.58 ***	0.30 ***	0.30 ***	0.30 ***	0.36 ***	-				
7. Distortion of Consequences	0.70 ***	0.50 ***	0.52 ***	0.46 ***	0.43 ***	0.34 ***	-			
8. Attribution of Blame	0.64 ***	0.41 ***	0.37 ***	0.49 ***	0.51 ***	0.29 ***	0.32 ***	-		
9. Dehumanization	0.63 ***	0.52 ***	0.37 ***	0.43 ***	0.37 ***	0.24 ***	0.38 ***	0.43 ***	-	
10. Age	0.17 ***	0.17 ***	0.07	0.13 **	0.11 *	0.09	0.04	0.18 ***	0.16 **	-
11. SES	−0.09	−0.09	−0.05	−0.02	−0.06	−0.07	−0.07	−0.15 **	−0.10 *	0.23 ***

* *p* < 0.05, ** *p* < 0.01, *** *p* < 0.001.

**Table 5:** Multinomial logistic regression.

Group	Predictor	B	SE	*p*	OR	95% CI
SNV–Control	Constant	−12.48	2.90	<0.001	3.78 × 10^−16^	[1.28 × 10^−8^–0.00]
	Male	0.61	0.31	0.05	1.84	[0.98–3.46]
	Age	0.89	0.18	<0.001	2.43	[1.69–3.51]
	SES	−0.82	0.15	<0.001	0.43	[0.32–0.59]
	MDDS Scale	0.01	0.01	0.22	1.01	[0.99–1.03]
	Moral Justification	0.12	0.07	0.10	1.13	[0.97–1.32]
	Euphemistic Language	−0.24	0.09	0.01	0.78	[0.64–0.94]
	Advantageous Comparison	0.00	0.11	0.98	1.00	[0.79–1.26]
	Displacement of Responsibility	0.05	0.07	0.49	1.05	[0.91–1.21]
	Diffusion of Responsibility	0.02	0.05	0.71	1.02	[0.91–1.13]
	Distortion of Consequences	0.03	0.07	0.59	1.03	[0.90–1.19]
	Attribution of Blame	0.09	0.09	0.34	1.09	[0.90–1.33]
	Dehumanization	0.01	0.07	0.84	1.01	[0.87–1.18]
CRSA–Control	Constant	−25.30	3.57	<0.001	0.02 × 10^−11^	[9.17 × 10^−15^–1.13 × 10^−8^]
	Male	2.57	0.44	<0.001	13.1	[5.48–31.6]
	Age	1.56	0.22	<0.001	4.78	[3.08–7.41]
	SES	−1.18	0.20	<0.001	0.30	[0.20–0.46]
	MDDS Scale	0.03	0.01	0.005	1.03	[1.01–1.06]
	Moral Justification	0.19	0.09	0.03	1.21	[1.01–1.45]
	Euphemistic Language	−0.31	0.10	0.004	0.72	[0.58–0.90]
	Advantageous Comparison	0.20	0.13	0.11	1.23	[0.95–1.59]
	Displacement of Responsibility	0.04	0.08	0.62	1.04	[0.88–1.23]
	Diffusion of Responsibility	−0.03	0.06	0.60	0.96	[0.84–1.10]
	Distortion of Consequences	4.20 × 10^−4^	0.09	0.99	1.00	[0.83–1.19]
	Attribution of Blame	0.19	0.10	0.07	1.21	[0.98–1.51]
	Dehumanization	0.04	0.08	0.63	1.04	[0.87–1.24]

**Table 6:** One-factor ANOVA.

	X^2^	gl	*p*	ε2
MDDS Total Scale	25.37	2	<0.001	0.06
Moral Justification	27.60	2	<0.001	0.07
Euphemistic Language	2.12	2	0.34	0.00
Advantageous Comparison	27.07	2	<0.001	0.07
Displacement of Responsibility	12.57	2	<0.01	0.03
Diffusion of Responsibility	3.05	2	0.21	0.00
Distortion of Consequences	6.49	2	0.05	0.01
Attribution of Blame	25.47	2	<0.001	0.07
Dehumanization	16.60	2	<0.001	0.04

**Table 7:** Two-to-two Dwass–Steel–Critchlow–Fligner comparisons.

MDDS Total Scale			Moral Justification		Advantageous Comparison		Displacement of Responsibility		Attribution of Blame		Dehumanization	
	W	*p*	W	*p*	W	*p*	W	*p*	W	*p*	W	*p*
Control–SNV	3.45 *	0.03 *	2.93	0.09	1.51	0.53	2.81	0.11	3.65	0.02 *	3.66	0.02 *
Control–CRSA	6.79 ***	<0.001 ***	7.08	<0.001 ***	6.83	<0.001 ***	4.83	0.02 *	6.74	<0.001 ***	5.50 ***	<0.001 ***
SNV–CRSA	4.27	<0.01 ***	4.91	0.02 **	5.52	<0.001 ***	2.57	0.16	4.30	<0.01 **	2.63	0.51

* *p* < 0.05, ** *p* < 0.01, *** *p* < 0.001.

## Data Availability

The data are not publicly available due to due to privacy concerns.
